# A comparative study of job satisfaction among nurses, psychologists/psychotherapists and social workers working in Quebec mental health teams

**DOI:** 10.1186/s12912-017-0255-x

**Published:** 2017-11-15

**Authors:** Marie-Josée Fleury, Guy Grenier, Jean-Marie Bamvita

**Affiliations:** 1 Department of Psychiatry, McGill University, Montreal, Canada; 2 Douglas Mental Health University Institute Research Centre, 6875 LaSalle Blvd. Montreal, Quebec, H4H 1R3 Canada

**Keywords:** Job satisfaction, Nurses, Social workers, Psychologists/psychotherapists, Team processes, Team emergent states, Team attributes, Socio-professional characteristics

## Abstract

**Background:**

This study identified multiple socio-professional and team effectiveness variables, based on the Input-Mediator-Output-Input (IMOI) model, and tested their associations with job satisfaction for three categories of mental health professionals (nurses, psychologists/psychotherapists, and social workers).

**Methods:**

Job satisfaction was assessed with the Job Satisfaction Survey. Independent variables were classified into four categories: 1) Socio-professional Characteristics; 2) Team Attributes; 3) Team Processes; and 4) Team Emergent States. Variables were entered successively, by category, into a hierarchical regression model.

**Results:**

Team Processes contributed the greatest number of variables to job satisfaction among all professional groups, including team support which was the only significant variable common to all three types of professionals. Greater involvement in the decision-making process, and lower levels of team conflict (Team Processes) were associated with job satisfaction among nurses and social workers. Lower seniority on team (Socio-professional Characteristics), and team collaboration (Team Processes) were associated with job satisfaction among nurses, as was belief in the advantages of interdisciplinary collaboration (Team Emergent States) among psychologists. Knowledge sharing (Team Processes) and affective commitment to the team (Team Emergent States) were associated with job satisfaction among social workers.

**Conclusions:**

Results suggest the need for mental health decision-makers and team managers to offer adequate support to mental health professionals, to involve nurses and social workers in the decision-making process, and implement procedures and mechanisms favourable to the prevention or resolution of team conflict with a view toward increasing job satisfaction among mental health professionals.

## Background

Studies have found that mental health professionals are particularly affected by stressful situations as compared with health care professionals working in other fields [1–3]. Among nurses, for example, the prevalence of burnout is significantly higher for those working in mental health services [3–5], which may explain why nursing students tend to pursue careers in other healthcare fields [6]. For example, a comparative study of nurses in Iranian hospitals found that 47% of those working in psychiatry wards met the criteria for burnout, versus 29% in burn units, 17% in surgery and 7% in internal medicine [7]. Stress was also identified as high among clinical psychologists, especially for those with less experience [1]. As well, a comparative study of 203 psychologists working in a variety of public institutions revealed that burnout and job dissatisfaction were significantly higher among psychologists from correctional institutions and public mental health hospitals [8]. For their part, mental health social workers have been identified as particularly affected by stress and burnout [3] due to their shifting role in multidisciplinary teams, which puts them in competition with other professionals [1]. One study that assessed levels of burnout and job satisfaction among 200 mental health social workers from the New York metropolitan area found that burnout affected 57% of them [9]. A literature review regarding burnout among mental health social workers found an association with certain aspects of work organization [10]. Other research suggests that psychiatrists have higher rates of suicide, drug/alcohol use and mental health disorders than other medical professionals [3, 11] due to a lack of administrative support and difficulties in dealing with troubled patients [1]. Finally, occupational therapists working in mental health were found to suffer from higher levels of stress than their counterparts whose patients had physical disabilities [1, 12].

The stressors affecting mental health professionals emanate from a wide range of sources, including confrontation with violent, aggressive or suicidal patients, challenging interactions with other professionals, heavy workloads and administrative responsibilities, lack of resources, inappropriate referrals, absence of positive feedback, low pay, poor work environment and lack of supervision [2]. Moreover, mental health professionals are more stigmatized and have lower professional prestige than other health care professionals [2, 13]. Chronic work-related stress may trigger not only burnout among mental health professionals, but also absenteeism related to physical illnesses, mental health problems or alcohol/drug abuse, as well as increased risk of professional errors. Moreover, these conditions are further associated with low levels of job satisfaction, as well as high staff turnover [14].

Since the 2000s, most industrial countries have reformed their mental health systems, creating increased uncertainty for mental health professionals [15]. This occurs, in part, because of the challenges of meeting new standards in mental health that increasingly focus on providing time-limited, results-oriented and more cost-effective treatments [1]; but also because mental health professionals have had to deal with new practice guidelines that reduce their professional autonomy, while requiring greater management skills [16]. Mental health care increasingly involves multidisciplinary collaboration where colleagues who do not necessarily share beliefs, values and practices [17]. These exchanges may run the risk of creating, or intensifying, job dissatisfaction in situations where collaboration raises doubts about one’s competence or ability to provide effective services [16]. On a more global scale, mental health professionals working in the public health care systems of neoliberal countries are living under “liquid modernity”, a phase in the evolution of capitalism characterized by heightened uncertainty and instability, with deleterious effects on workplace environments in many fields, including health care. [18, 19]. Previously, nurses and other health care workers were trained, and tended to remain in single institutions throughout their careers, which allowed them to invest in long-term relationships with other staff, patients and families [19]. By contrast, current health care trends are marked by atomized roles, the routinisation and mechanisation of practices, unstable employment and high levels of staff turnover as nurses, and other professionals, turn to the open market for job opportunities. This has led to a certain disengagement among health workers from their work, and fosters the dehumanization of health care [19].

Job satisfaction is defined as the “overall assessment of positive emotions” that a worker has to his job [8]. Considering that the hours spent at work represent a very significant part of daily life for most people, job satisfaction is a major determinant of quality of life, in terms of happiness as well as mental and physical health [20]. Job satisfaction may, in fact, be considered “a general indicator of work-related quality of life” [13], which suggests why job satisfaction is a primary outcome variable in mental health research. Dissatisfied professionals may have a negative influence on colleagues, damaging the overall working environment [14]. They are also less likely to convey empathy, or engage in positive interactions, with clients [13, 14], suggesting negative implications for client satisfaction with mental health services [13, 14, 21]. Absenteeism and staff turnover due to burnout and job dissatisfaction have the further effect of disrupting established therapeutic relationships between professionals and clients [14, 22] as well as the quality and continuity of service delivery [14, 23]. Staff turnover resulting from high levels of job dissatisfaction also poses important financial hardships for the health care system, as hiring and training new staff are costly undertakings [23].

Among the tools required by mental health care managers responsible for providing high quality services and continuity of care, an accurate and clear understanding of variables associated with job satisfaction is crucial. Job satisfaction has been associated with socio-professional characteristics, team attributes, and relationships among team members [4]. The Input-Mediator-Outcomes-Input (IMOI) Model [24] conceptualizes job satisfaction, and other outcomes reflecting the quality of teamwork, as influenced by team processes (i.e., actions affecting teamwork, such as team support or collaboration), and team emergent states (i.e. motivation, cognition or emotions resulting from team involvement, such as affective commitment to the team or belief in the advantages of interdisciplinary collaboration) [25]. Team processes and team emergent states are in turn influenced by team attributes (e.g. composition, setting) and individual socio-professional characteristics (e.g. age, gender, type of profession) [24].

The main team processes associated with job satisfaction among mental health professionals include: team support [13], autonomy [26], collaboration [27], involvement in decision-making processes, informational self-efficacy, or a personal belief in the “ability to accomplish a task or cope with environmental demands” [28], and low levels of team conflict [29]. Previous studies have also discovered associations between job satisfaction and team emergent states such as team climate [30], affective commitment to the team [29], and trust [29]. Regarding team attributes, some differences were found concerning the influence of team composition on job satisfaction. While the presence of an overly large number of professionals may increase the risk of team conflict and member dissatisfaction [31], staff shortages may increase tasks and caseloads for individual team members, with the opposite effect of hindering job satisfaction [32]. Some studies have suggested that mental health nurses working in the community enjoy high levels of job satisfaction [33–35] due to their greater autonomy [34]; whereas other studies have found, to the contrary, that job satisfaction was greater among hospital-based nurses due to the support provided by their organization [36]. Regarding socio-professional characteristics, two studies identified a negative association between age and years of experience, respectively, and job satisfaction [5, 37]. Yet type of profession was identified as the main professional characteristic related to job satisfaction, with nurses [32] and social workers [38] usually less satisfied than physicians or psychiatrists [15, 27, 39, 40].

Other studies have assessed job satisfaction among mental health professionals, both in general, and specifically among mental health nurses; yet few have examined variables associated with job satisfaction for different types of mental health professionals [40]. Moreover, job satisfaction is usually assessed in relation to socio-professional characteristics, including various dimensions of burnout (emotional exhaustion, depersonalization, personal accomplishment), objective employment conditions (e.g. social benefits, salary), as well as perceived organizational conditions (e.g. social support, potential for promotion) [21]. Importantly, dimensions related to teamwork (team processes and team emergent states) have rarely been assessed. Moreover, to the best of our knowledge, the respective contributions of socio-professional characteristics, team attributes, team processes and team emergent states to job satisfaction among various types of mental health professionals working both in primary care and in specialized mental services have not yet been examined.

This study aimed to identify variables associated with job satisfaction among three categories of mental health professionals (nurses, psychologists/psychotherapists, and social workers) and to investigate the relative contribution of socio-professional characteristics, team attributes, team processes and team emergent states to job satisfaction for each type of professional.

## Methods

### Study design and sample

The sample included mental health professionals from four local health service networks located in the province of Quebec (Canada). These networks were selected for diversity in terms of geographic area (urban or semi-urban), population (e.g. percentage of population with low income) and services offered (e.g. presence of a psychiatric hospital, or not). A list of mental health professionals eligible for the study was provided by team managers from the four networks. The inclusion criteria stipulated that professionals had to be members of a mental health team with at least three members, representing at least two distinct disciplines. In terms of specific professional affiliation, they could be nurses, social workers, psychologists/psychotherapists, physicians, pharmacists, or others (occupational therapists, technicians, clerks). In categories where the numbers were low (e.g. physicians and pharmacists) or job descriptions too diffuse, job satisfaction was not assessed. The research ethics board of the Douglas Mental Health University Institute approved the multi-site study protocol.

### Data collection, variables, conceptual framework, and standardized scales

Data collection took place between May 2013 and June 2014. A total of 466 mental health professionals were invited to complete a self-administrated questionnaire that included standardized scales related to diverse aspects of teamwork (e.g. team support, team conflicts), and questions regarding individual socio-professional characteristics (e.g. age, employment status). Team managers for all teams in the selected networks (*n* = 49) were approached to complete a second questionnaire covering client profiles (e.g. diagnoses), team characteristics (e.g. team size, composition), clinical activities (e.g. use of different clinical approaches), organizational culture, integration strategies used (e.g. service agreements), as well as frequency and satisfaction of interactions with others teams and organizations. Each participant signed a consent form.

Job satisfaction was assessed with the Job Satisfaction Survey [41]. This 20-item scale includes five sub-dimensions of job satisfaction: supervision, contingent reward, operating procedures, co-workers, and nature of the work. Items dealing with remuneration were excluded as they did not apply to the Quebec public health care system. Cronbach’s alpha on job satisfaction for the present study varied between 0.63 (co-workers) and 0.77 (contingent reward).

**Fig. 1:**
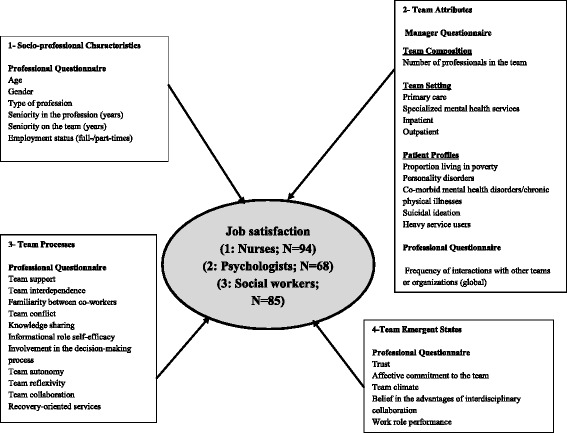
Conceptual Framework

Independent variables were classified into four categories, based on a conceptual framework (Fig. 1) inspired by the IMOI Model: 1) Socio-professional Characteristics; 2) Team Attributes; 3) Team Processes; and 4) Team Emergent States. Socio-professional Characteristics included six variables from the professional questionnaire: age, gender, type of profession, seniority in the profession, seniority on the team, and employment status (full- or part-time). Team Attributes included three variables from the manager questionnaire: team composition; team setting and patient profiles, as well as one variable from the professional questionnaire (frequency of interactions with other teams or organizations).

Team Processes included 11 variables, and Team Emergent states, five variables, all of which were assessed with standardized scales (Table 1). With the exception of team collaboration, informational self-efficacy, and belief in the advantages of interdisciplinary collaboration, where the instruments were originally developed in French, all standardized scales were translated from English to French, and validated.

**Table 1:** Description of Standardized Instruments Included in the Study

Measures and References	Description	Cronbach’s Alpha Coefficients from the Original Validation
Dependent Variable		
Job satisfaction [41]	20 items; 5 sub-dimensions	0.90
Independent Variables		
*For Team Processes:*		
Team support [56]	4 items	0.84–0.85
Team interdependence [57]	20 items	0.77–0.88
Familiarity between co-workers [58]	5 items	0.78–0.88
Team conflict [59]	9 items	0.93–0.94
Knowledge sharing [60]	5 items	0.93
Informational role self-efficacy [61]	5 items	0.93
Involvement in the decision-making process [62]	3 items	0.88
Team autonomy [62]	3 items	0.76
Team reflexivity [63]	3 items	0.79
Team collaboration [64]	14 items	0.77–0.91
Recovery-oriented services [65]	32 items; 5 sub-dimensions	0.76–0.90
*For Team Emergent States:*		
Trust [66]	4 items	0.90
Affective commitment to the team [52]	5 items	0.86–0.92
Team climate [67]	19 items; 4 sub-dimensions	0.60–0.84
Belief in the advantages of interdisciplinary collaboration [62]	5 items	0.92
Work role performance [68]	18 items	0.90

### Preliminary analyses

Analyses were conducted using SPSS, 24th edition. First, the database was screened for outliers and missing values, which were treated using multiple imputation techniques. This method consists of running multiple sets of regression analyses with variables pertaining to the same category (e.g., socio-demographic variables), revealing what would be the most likely responses from participants in place of the missing data based on how each individual responded to other questions. Frequency distributions for categorical variables, and central tendency measures (means, standard deviations) for continuous variables, were calculated. The dependent variable, job satisfaction, was normally distributed (Skewness: −0.037; Kurtosis: 0.332).

### Multilevel analyses

Due to the nested nature of the variables, analyses were performed to assess the need for taking into account the wider contextual level (i.e., teams), above the individual level. The intra-class correlation was calculated to assess homogeneity within teams and heterogeneity between teams. Using the Maximum likelihood method with random effects, the significance of clustering at the team level was estimated by means of Wald Z test.

### Hierarchical regression analysis

Associations between each independent variables and the dependent variable (job satisfaction) were assessed separately using ANOVA and t-tests, with Alpha set at 0.10. The independent variables found to be significantly associated with job satisfaction were used to build a hierarchical linear regression model, with Alpha set at 0.05. The four blocks of variables presented in Fig. 1 were entered successively, in order to assess the individual contribution of each set of variables, by block, and, more importantly, to estimate the contribution of the final model after controlling for all previously introduced variables. For the first block, the Backward Elimination technique was used so that only variables significantly associated with job satisfaction were retained in the model, using an Alpha value of 0.10 for elimination. For the second model, variables significantly associated with job satisfaction in the first model were introduced by the Forced Entry method; and variables pertaining to the second block were introduced using Backward Elimination. Successive rounds of variables were introduced similarly using the Forced Entry and Backward Elimination techniques to build the third and the fourth models. The explained variance was estimated for each model, along with the goodness-of-fit (ANOVA F test and *p* value).

## Results

### Sample

A total of 315 mental health professionals participated in the study, for a response rate of 68%. No significant differences were found between participant and non-participant mental health professionals with respect to distributions for type of team [χ 2 (1, *N* = 466) = 0.79; *p* = 0.68] and gender [χ 2 (1, N = 466) = 0.03; *p* = 0.87]. In terms of socio-demographic profile, the mean age of participants was 43 years, with a larger proportion of women (70%) than men. Average seniority within the profession was nine years, and within the team three years. Most were working full-time (78%), with a much smaller proportion working part-time (22%). Almost a third of participants worked in primary care teams (32%), whereas more than half of the remaining participants worked in outpatient specialized mental health care teams (56%), and the others in inpatient specialized mental health care teams (12%). Regarding type of profession, the most prevalent category consisted of nurses (*N* = 94; 30%), followed by social workers (*N* = 85; 27%), and psychologists/psychotherapists (*N* = 68; 22%), which together represented 79% of the total sample. There were no significant differences in job satisfaction scores among the three groups. Other mental health professionals (e.g. technicians, clerks) constituted 17% (*N* = 53) of the total sample, while physicians (including psychiatrists) and pharmacists represented only 5% (*N* = 15). Concerning clients of participating mental health professionals, 37% had severe mental health disorders (e.g., bipolar and psychotic disorders), 21% personality disorders, and 20% suicidal ideation. These and other participant characteristics are presented in Table 2.

**Table 2:** Descriptive Statistics

			Global (*N* = 313)				Nurses (*N* = 94)		Psychologists/Psychotherapists (*N* = 68)		Social workers (*N* = 85)	
			Min	Max	N/Mean	%/SD	N/Mean	%/SD	N/Mean	%/SD	N/Mean	%/SD
1. Socio-Professional Characteristics	Age		24.0	68.0	43.3	10.5	44.3	9.3	40.0	9.7	41.9	11.3
	Gender	female			219	69.5	61	64.9	53	77.9	59	69.4
		male			96	30.5	33	35.1	15	22.1	26	30.6
	Professions	Nurses			94	29.8						
		Physicians & Pharmacists			15	4.8						
		Psychologists & Psychotherapists			68	21.6						
		Social workers			85	27.0						
		Other mental health professionals			53	16.8						
	Seniority in the profession (in years)		0.0	38.0	9.0	10.8	8.8	10.8	6.1	8.6	8.4	9.7
	Seniority on team (in years)		0.0	27.0	3.1	4.6	2.9	4.5	2.5	3.6	2.9	4.0
	Employment status	Full time			246	78.1	89	94.7	22	44.9	69	87.3
		Part time			69	21.9	5	5.3	27	55.1	10	12.7
2. Team Attributes	Clients	Personality disorders	2.0	90.0	30.6	21.3	27.6	18.6	38.4	24.6	32.8	20.4
		Co-morbid mental health disorders/chronic physical illnesses	2.0	93.0	34.4	21.6	37.4	22.6	27.8	21.2	31.5	17.8
		Suicidal ideation	0.0	95.0	27.9	19.9	25.0	18.3	28.5	24.6	30.1	17.7
		Health care service high-users	0.0	90.0	26.0	24.0	26.7	25.4	19.6	21.5	26.4	22.4
		Severe mental health disorders (bipolar disorder and other psychoses)	2.0	149.9	66.9	37.2	69.6	34.2	52.1	41.8	62.6	34.8
	Settings	Primary care teams			101	32.1	16	17.0	29	59.2	38	48.1
		Outpatient specialized mental health care teams			176	55.9	60	63.8	19	38.8	38	48.1
		Inpatient specialized mental health care teams			38	12.1	18	19.1	1	2.0	3	3.8
	Number of professionals in the team		3.0	16.0	8.0	3.5	8.0	3.5	7.6	3.0	8.1	3.2
	Proportion of patients living in poverty		5.0	23.3	14.2	8.1	15.7	7.9	14.1	8.3	14.7	8.4
	Frequency of Interactions with other teams or organizations		32.0	204.0	95.4	31.3	103.6	31.0	77.3	24.5	99.3	32.4
3. Team Processes		Team support	1.0	7.0	4.8	1.2	4.8	1.3	4.7	1.2	4.9	0.9
	Team interdependence		4.6	20.7	13.7	3.1	13.8	3.1	12.4	3.1	13.1	3.3
	Familiarity between co-workers		1.6	7.0	5.4	0.9	5.5	1.0	5.0	1.2	5.4	0.7
		Team conflict	3.0	21.0	9.0	2.9	9.3	3.4	8.6	2.0	9.1	3.2
		Knowledge sharing	1.8	7.0	5.7	0.9	5.8	1.0	5.6	0.9	5.6	0.9
	Informational role self-efficacy		16.0	100.0	81.1	14.4	80.7	16.2	82.4	11.8	82.1	15.1
	Involvement in the decision-making process		1.0	7.0	5.0	1.4	5.1	1.3	4.7	1.4	4.8	1.5
		Team autonomy	1.0	7.0	4.9	1.3	5.1	1.1	4.7	1.5	4.7	1.3
		Team reflexivity	1.0	7.0	4.6	1.2	4.8	1.2	4.3	1.3	4.3	1.3
	Team collaboration		8.5	28.0	19.3	3.8	19.7	4.4	19.0	3.8	18.5	3.6
	Recovery-oriented services		2.2	6.8	5.1	0.7	5.1	0.8	5.1	0.5	5.1	0.6
4. Team Emergent States		Trust	1.0	7.0	5.2	1.2	5.4	1.1	5.2	1.1	5.1	1.3
	Affective commitment to the team		1.0	7.0	4.9	1.2	4.9	1.4	4.9	1.2	4.6	1.3
	Team climate	Total score	7.9	27.8	20.5	3.4	20.7	3.6	20.4	3.2	19.6	3.6
		Participatory safety	1.0	7.0	5.2	0.9	5.2	1.0	5.1	0.9	5.0	1.1
		Support for innovation	1.0	7.0	5.1	1.0	5.3	1.1	5.1	0.9	4.9	1.1
		Vision orientation	1.5	7.0	5.1	1.0	5.2	1.1	5.2	0.8	4.8	1.0
		Vision orientation	1.5	7.0	5.1	1.0	5.1	1.1	4.9	1.1	4.9	1.0
	Belief in the advantages of interdisciplinary collaboration		3.0	7.0	6.2	0.7	6.3	0.7	6.3	0.7	6.1	0.8
	Work role performance		23.7	42.0	34.6	3.3	35.4	3.4	33.8	3.1	34.2	3.3
		Job satisfaction	11.3	35.0	24.8	3.6	24.2	3.8	24.7	3.2	24.8	3.6

Forty-one of the 49 team managers recruited to the study participated, for an 84% response rate. No significant differences were found between participant and non-participant team managers on gender (Pearson chi-square = .966; df = 1; Fisher’s exact test two-sided *p* = .663); or type of team (Pearson chi-square = 1.861; df = 1; Fisher’s exact test two-sided *p* = .245). Among participating managers, 71% were female, and 62% were members of specialized mental health care teams versus 38% in primary care teams. The mean age of participating team managers was 44, and mean seniority on team was four years.

### Multilevel analysis

The 315 mental health professionals represented 49 teams. These teams, which had an average of 6 members each (ranging from 3 to 16), were clustered into 9 types of team (teams in specialized mental health services: hospital units, day hospitals, assertive community treatment programs, specialized outpatient clinics, rehabilitation programs; and teams in primary care services: primary care teams, evaluation units, local community service centres, and intensive case management programs), with a mean of 35 participants per group (ranging from 30 to 55). Intra-class correlation (ICC) was calculated on variables of interest, and found to be elevated (84%). The effect of clustering was then calculated using Maximum Likelihood ratio with random effects, and found to be non-significant (Wald Z = 1.377; *P* = 168), meaning that the nested nature of the data and clustering into groups (or types of team) did not bring about any added value to the individual-level model.

### Hierarchical regression analysis

Variables associated with job satisfaction among nurses are presented in Table 3. From the Socio-professional Characteristics block, only one variable was retained: seniority on the team, which was negatively associated with job satisfaction, suggesting that younger nurses were more satisfied with their jobs than older nurses. A single variable from the Team Attributes block, frequency of interactions with other teams or organizations, was positively, but marginally, associated. The third block, Team Processes, produced three positively associated variables (team collaboration, involvement in the decision-making process, and team support) and one negative association for team conflict. In addition to these variables, one variable previously retained in the model, seniority on the team (Socio-professional Characteristics), remained negatively, but marginally, associated in the regression model. The final model explained 49% of the total variance.

**Table 3:** Variables associated with job satisfaction among nurses in mental health services: Hierarchical Linear Regression model (N = 94)

	Model 1: Professional Characteristics		Model 2: Team Attributes		Model 3: Team Processes						
	*SC β	P			*SC β	t	P	95.0% CI for B		Collinearity Statistics	
								Lower Bound	Upper Bound	Tolerance	VIF
(Constant)		<0.001		<0.001		8.555	<0.001	12.519	20.097		
Seniority on the team (years)	−0.229	0.026	−0.218	0.033	−0.161	−1.971	0.052	−0.270	0.001	0.882	1.134
Frequency of interactions with other teams or organizations			0.184	0.071	−0.044	−0.525	0.601	−0.026	0.015	0.824	1.214
Team collaboration					0.265	2.512	0.014	0.047	0.404	0.532	1.880
Team conflict					−0.259	−3.177	0.002	−0.464	−0.107	0.891	1.123
Involvement in the decision-making process					0.260	2.813	0.006	0.224	1.302	0.692	1.445
Team support					0.224	2.513	0.014	0.135	1.154	0.746	1.340
Goodness-of-fit:											
ANOVA: F	5.108		4.292		13.692						
ANOVA: P	0.026		0.017		<0.001						
Total variance explained: R^2^:	0.053		0.086		0.486						

*SC β: Standardized Coefficients Beta

Variables associated with job satisfaction among psychologists/psychotherapists are presented in Table 4. Only two variables were significant: team support from the Team Processes block, and belief in the advantages of interdisciplinary collaboration, from the Team Emergent States block. Both variables were positively associated with job satisfaction, and together contributed 41% to the total variance.

**Table 4:** Variables associated with job satisfaction among psychologists/psychotherapists in mental health services: Hierarchical Linear Regression

	Model 1: Team Processes		Model 2: Team Emergent States						
			*SC β	t	P	95.0% CI for B		Collinearity Statistics	
						Lower Bound	Upper Bound	Tolerance	VIF
(Constant)		<0.001		2.837	0.007	2.959	17.425		
Team support	0.595	<0.001	0.585	5.152	<0.001	0.988	2.256	0.998	1.002
Belief in the advantages of interdisciplinary collaboration			0.234	2.058	0.045	0.024	2.139	0.998	1.002
Goodness-of-fit:									
ANOVA: F	25.795		15.904						
ANOVA: P	<0.001		<0.001						
Total variance explained: R^2^:	0.354		0.409						

*SC β: Standardized Coefficients Beta

Variables associated with job satisfaction among social workers are presented in Table 5 **.** The second block, Team Attributes, contributed one variable from the Patient Profiles sub-category, namely personality disorders, which was negatively associated with job satisfaction, suggesting that social workers dealing with a higher proportion of patients with personality disorders were more dissatisfied with their jobs. Four variables from the third block, Team Processes, were also significant, including three positive associations (team support, knowledge sharing, and involvement in the decision-making process), and one negative association (team conflict) with the dependent variable. The fourth block, Team Emergent States, contributed one variable: affective commitment to the team, which was positively associated with job satisfaction. The four Team Processes variables previously retained in the model remained significant, but marginally so; whereas knowledge sharing, while personality disorders in the Patients Profiles subcategory were not retained. The final model explained 65% of the total variance. A collinearity diagnosis for the three hierarchical models, using Tolerance and variance inflation factor (VIF), demonstrated that the variables were not overly correlated.

**Table 5:** Variables associated with job satisfaction among social workers in mental health services: Hierarchical Linear Regression model (N = 85)

	Model 1: Team Attributes		Model 2: Team Processes		Model 3: Team Emergent States						
	*SC β	P			*SC β	t	P	95.0% CI for B		Collinearity Statistics	
								Lower Bound	Upper Bound	Tolerance	VIF
(Constant)		<0.001		<0.001		7.432	<0.001	11.266	19.524		
Personality disorders	−0.344	0.002	−0.135	0.087	−0.099	−1.311	0.194	−0.044	0.009	0.845	1.184
Team support			0.323	0.001	0.316	3.552	0.001	0.530	1.885	0.612	1.634
Team conflict			−0.468	<0.001	−0.430	−5.843	<0.001	−0.661	−0.325	0.897	1.115
Knowledge sharing			0.206	0.017	0.163	1.968	0.053	−0.008	1.302	0.710	1.408
Involvement in the decision-making process			0.229	0.027	0.196	1.994	0.050	<0.001	0.923	0.501	1.996
Affective commitment to the team					0.205	2.649	0.010	0.144	1.017	0.811	1.233
Goodness-of-fit:											
ANOVA: F	10.323		23.465		22.335						
ANOVA: P	0.002		<0.001		<0.001						
Total variance explained: R^2^:	0.118		0.616		0.651						

*SC β: Standardized Coefficients Beta

## Discussion

Most variables significantly associated with job satisfaction among nurses, psychologists/psychotherapists and social workers in the present study emanated from the Team Processes block, whereas the respective contributions of Professional Characteristics, Team Attributes and Team Emergent States ranged from minimal to none. Team Processes provided 80% of the variables associated with job satisfaction among both nurses and social workers, and 50% of variables in the psychologists/psychotherapists group.

The only variable associated with job satisfaction among nurses, psychologists/psychotherapists, and social workers was team support, which may take the form of either instrumental or emotional support [20]. Support from supervisors or co-workers is important in human services, but in mental health organizations more especially, considering the high level of work-related stress experienced by professionals in this field [13]. More specifically, supervisors may control dimensions related to the work environment that may contribute to a more positive working climate and combat emotional exhaustion among professionals frequently exposed to complex cases [22].

A low level of team conflict was the main contributor to job satisfaction among social workers and nurses, yet had no relationship to job satisfaction among psychologists/psychotherapists. Social workers and nurses are particularly affected by task conflicts and role ambiguity, according to the literature, as their functions are often misunderstood by other professionals [5, 10, 34, 42]. Moreover, these two professionals groups often need to deal with the contradictory expectations of clients, relatives, other professionals, and supervisors, which serves to increase conflict situations [10, 43].

The finding that job satisfaction was influenced by involvement in the decision-making process among nurses and social workers seemed to suggest an awareness that their expertise was being acknowledged by other mental health professionals. According to Lichtenstein et al. [39], the level of involvement in the decision-making process influences satisfaction with professional autonomy as well as with relationships among team members. Participation in the decision-making process was also found to deepen the commitment of professionals to their respective teams [44, 45]. Yet some organizational cultures are more likely to value the empowerment and participation of professionals in the decision-making process than others. The clan culture, characterised by particular values such as loyalty, development, participation, and staff empowerment, as well as flexibility and internal focus, is the prime example [46]. This type of organizational culture, more implemented usually in smaller organizations, would have a more positive effect on job satisfaction for example in primary care teams within a mental health context [47].

The association between greater team collaboration (Team Processes) and job satisfaction among nurses has been previously revealed [6, 48]. Effective collaboration reduces risk of role ambiguity and task conflicts [33], while facilitating trust among team members [49].

It stands to reason that involvement in the decision-making process provides social workers with greater opportunities to share information, methods and experiences with other mental health professionals [50]. The association between knowledge sharing and job satisfaction among social workers may be explained by the fact that knowledge sharing increases self-perceptions of competence [16]. Previous studies reported higher levels of job satisfaction among social workers with higher perceived self-efficacy [51].

Team Emergent States variables contributed to job satisfaction among psychologists/psychotherapists and social workers, but not among nurses. Affective commitment to the team was the third strongest variable associated with job satisfaction among social workers. It is possible that this Emergent State variable was, in turn, strongly related to both knowledge sharing and involvement in the decision-making process on teams. According to the literature, professionals highly committed to their teams were more likely to enjoy shared values and less likely to quit their jobs [17, 21, 52]. Furthermore, social workers who perceived themselves as capable of performing well were more likely to have a better partnership with their supervisors and co-workers [16].

Regarding psychologists/psychotherapists, job satisfaction was related to belief in the advantages of interdisciplinary collaboration. This suggests that the presence of psychologists/psychotherapists in mental health teams was based more on their belief in shared goals and responsibilities among mental health professionals [53] than on their affective commitment to other team members. This resulted perhaps from the well-known fact that psychologists/psychotherapists enjoy the highest levels of professional autonomy, including more opportunities than social workers or nurses to find jobs in other teams, or even other health care fields, when dissatisfied. Moreover, psychologists/psychotherapists have the highest professional status in mental health, second only to psychiatrists [39].

Professional Characteristics contributed to job satisfaction only among nurses. That is, nurses with lower seniority on teams were more satisfied with their jobs, which seems to contradict the literature. Previous studies found that younger mental health nurses had greater levels of stress, and higher risk of burnout; they were more likely to leave their jobs than those with more years of work experience [54]. In general, job satisfaction is higher among older professionals [55], mainly because they experience lower levels of depersonalization, a key dimension of burnout [13]. One explanation for our finding may be that relatively more younger nurses were hired to work in the newly created primary care mental health teams, and found this is a positive experience. Mental health nurses working in the community would also have higher levels of autonomy as especially compared with those working in inpatient mental health services [34].

Finally, no variables related to Team Attributes appeared in any of the three final models. Among social workers, the contribution of clients with personality disorders to job satisfaction became marginal after the introduction of Team Processes, and disappeared entirely after the introduction of Team Emergent States variables. This result suggests that positive Team Processes may neutralize the stress resulting from contacts with difficult clients such as the ones affected by personality disorders. Among nurses, the marginal association between the frequency of interactions with other teams or organizations and job satisfaction was also eliminated after the introduction of Team Process variables, suggesting that job satisfaction was not based on frequent contact with other teams or organizations but rather on harmonious processes within the teams.

This study has limitations that should be acknowledged. First, as the data were cross-sectional, we were unable to determine whether the various independent variables promoted job satisfaction, or vice versa. Second, the teams had to be clustered into team types for the purposes of computing interclass correlations, as many individual teams had insufficient numbers for this analysis. Third, we did not have data for certain variables known to be strongly associated with job satisfaction, such as caseload. Fourth, due to their low numbers in the initial sample, it was impossible to assess variables associated with job satisfaction among psychiatrists, general practitioners and occupational therapists. Finally, our results may not be generalized to other samples consisting of single professions, or to specific types of mental health teams.

## Conclusions

This study was original in assessing variables associated with job satisfaction for three categories of mental health professionals working in four different local health service networks, and in different types of mental health care teams, using a large number of standardized scales related to various dimensions of team effectiveness. The results reveal the very important contribution of team processes to job satisfaction among nurses, psychologists/psychotherapists and social workers alike.

The emergence of team support as the only variable associated with job satisfaction among all three categories of mental health professionals, confirms the importance of this variable for consideration by mental health decision makers and team managers in improving clinical practice. They need to offer adequate financial, material and social support to mental health professionals with a view toward improving both job satisfaction among professionals working on mental health teams and, more indirectly, the satisfaction of clients with respect to the services they receive. The results also indicate the major importance of involving all mental health professionals in the decision-making process, and of implementing procedures and mechanisms favourable to the prevention, or resolution, of team conflict in order to enhance job satisfaction, particularly among nurses and social workers as the most numerous professionals in mental health teams, as well as those generally responsible for providing frontline follow-up services to clients with active mental disorders.
